# Syphilis as Rare Cause of Pyogenic Liver Abscess

**DOI:** 10.3201/eid3107.250744

**Published:** 2025-07

**Authors:** Danielle Meyer, Michele Granada

**Affiliations:** Abbott Northwestern Hospital, Minneapolis, Minnesota, USA

**Keywords:** Syphilis, bacteria, pyogenic liver abscess, liver, *Treponema pallidum*, United States

## Abstract

Syphilis has a wide range of possible symptoms, making it difficult to diagnose. We report a rare case of liver abscess secondary to *Treponema pallidum* in a man in Minnesota, USA, who had well-controlled HIV infection. This case emphasizes the importance of appropriate screening for syphilis, especially in high-risk populations.

Syphilis is a sexually transmitted infection caused by the spirochete bacterium *Treponema pallidum*. Its diverse manifestations can make syphilis difficult to diagnose. The disease progresses through 4 main stages. The early phase begins with a localized skin lesion at the site of inoculation. If left untreated, hematogenous dissemination can lead to secondary syphilis, characterized by a diffuse maculopapular rash and systemic symptoms. Tertiary syphilis represents a later stage that can affect multiple organ systems. Involvement of the liver is uncommon and can result in syphilitic hepatitis or hepatic gummas, granulomatous soft tissue lesions with central necrosis. We report an exceptionally rare case of syphilitic liver abscess confirmed with 16s rDNA PCR.

A 52-year-old man in Minnesota, USA, with a history of well-controlled HIV infection (CD4 count 767) on a regimen of dolutegravir/rilpivirine sought treatment for symptoms including 3 months of diarrhea and bilateral ankle edema. Two weeks before his initial visit and at the request of his healthcare provider, the man provided blood samples for laboratory assessment, which revealed elevated levels of alkaline phosphatase (ALP [557 IU/L; reference range 35–144 IU/L]), aspartate transaminase (AST [67 IU/L; reference range 10–35 IU/L]), and alanine aminotransferase (ALT [160 IU/L; reference range 9–46 IU/L]). Repeat laboratory results 1 week later showed persistently elevated ALP (484 IU/L), AST (58 IU/L), and ALT (88 IU/L). An abdomen ultrasound demonstrated hepatic steatosis.

Physical examination was notable for edema in bilateral lower extremities. Blood analysis revealed further elevation of ALP (586 IU/L), AST (68 IU/L), and ALT (102 IU/L). Abdomen and pelvis computed tomography with contrast (Figure, panel A) identified a 3.8 × 2.3 × 3.3-cm peripheral mass in the right lobe of the liver, and he was subsequently admitted to the hospital for further evaluation.

**Figure:**
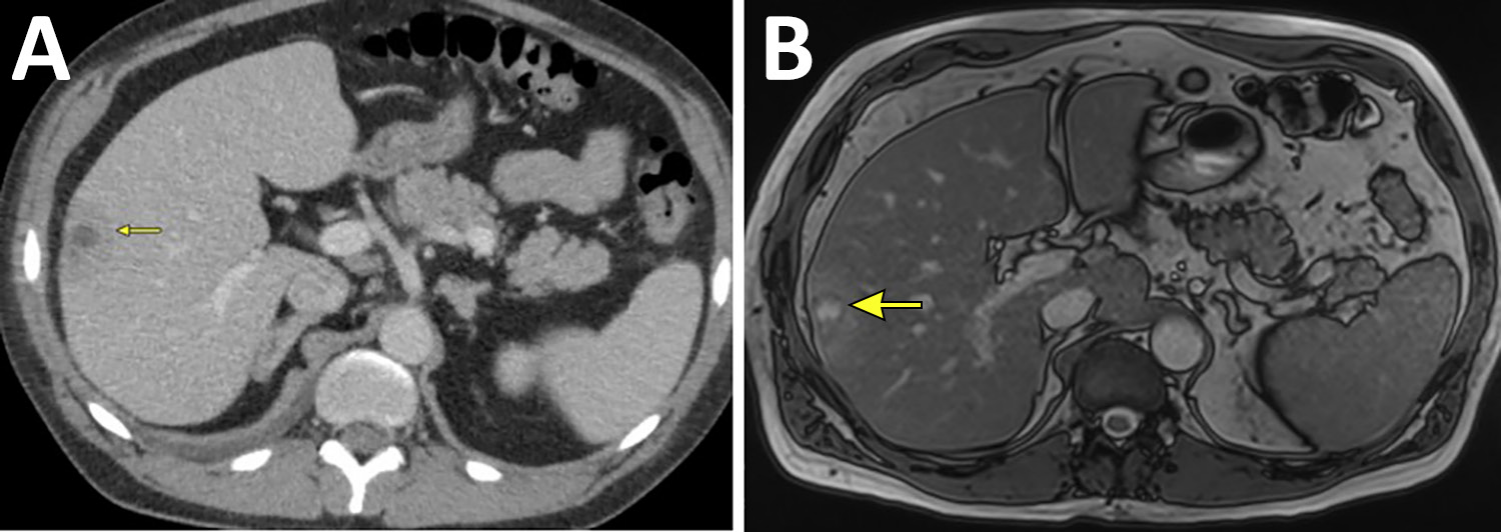
Imaging findings from a study of syphilis as a rare cause of pyogenic liver abscess in an HIV-positive man in Minnesota, USA. Computed tomography (A) and magnetic resonance imaging (B) of the abdomen and pelvis show rim-enhancing lesion (yellow arrows) in segments 5 and 6 of the liver.

Viral hepatitis serology test results were negative. A stool multiplex PCR test was positive for *Shigella*. Abdominal magnetic resonance imaging (Figure, panel B) confirmed a 2.4-cm rim-enhancing lesion in the lateral aspect of segments 5 and 6 of the liver. Ultrasound-guided aspiration of the liver lesion yielded 1 mL of yellow, purulent fluid and provided 4 core biopsy samples. The patient elected to leave the hospital early and was discharged home with a 4-week course of oral ciprofloxacin (500 mg 2×/d) and metronidazole (500 mg 2×/d) for empiric coverage of possible hepatic abscess as well as coverage for shigellosis.

One week later, liver abscess cultures were negative. Pathology revealed a benign abscess, background intact liver parenchyma, and negative results for neoplasia. We requested 16s rDNA and 28s rDNA PCR tests on liver tissue. The man visited his healthcare provider for routine follow-up. Screening for *T. pallidum* antibody demonstrated reactivity, and rapid plasma reagin (RPR) testing revealed elevated results (1:32). Previous RPR titers were negative. The man received 1 doseof intramuscular benzathine penicillin (2.4 million units). The following day, 16s rDNA PCR testing of a liver tissue sample was positive for *T. pallidum*. The man subsequently completed 2 additional weekly doses of benzathine penicillin. One week after completing treatment, his RPR test result was 1:64, and tests measuring his hepatic function and C-reactive protein were within reference ranges. Computed tomography of his abdomen and pelvis showed a reduction in the hepatic lesion to 1.1 × 0.9 cm, a marked improvement in size and appearance and consistent with healing.

Syphilis resulting in abscess is rare. Few cases have been reported, including abscess associated with the pituitary gland (*1*), lungs (*2*), scrotum (*3*,*4*), and lymph nodes (*5*). Three cases (*6*,*7*) in the 1920s described what were thought to be liver abscesses caused by syphilis. Researchers presumed their findings based on symptoms and examination findings suggestive of liver abscess in the setting of positive serologic results and symptomatic improvement with treatment. However, the suspected abscesses were neither drained nor confirmed with further testing.

In this patient, the clinical picture was complicated by shigellosis, which was the likely etiology of his diarrhea. He did not have additional symptoms suggestive of hepatic abscess. He did have persistently elevated liver enzymes, particularly ALP. Liver function tests are abnormal in up to 39% of patients diagnosed with early syphilis, most of whom are asymptomatic (*8*). Furthermore, an increase in RPR titers immediately after treatment is not uncommon in early stages of treatment and is not indicative of treatment failure. This patient had a marked decrease in abscess size and resolution of transaminitis. Our question initially was if this was syphilitic gumma of tertiary syphilis, but given the absence of granulomatous inflammation on pathology, this man’s case more likely demonstrated secondary syphilis with syphilitic hepatitis. 

In its early stage, syphilitic hepatitis can be asymptomatic with a disproportionally elevated ALP in the setting of secondary syphilis, resulting in rare occurrences of abscesses in the liver. It can also cause hepatic inflammatory masses in HIV-positive men who have sex with men (*9*). This case demonstrates a rare and diagnostically complex clinical manifestation of secondary syphilis, emphasizing the importance of appropriate screening for syphilis, especially in high-risk populations whose laboratory and imaging assessments reveal elevated liver enzymes and hepatic mass lesions.
